# Reverse Translation in Tuberculosis: Neutrophils Provide Clues for Understanding Development of Active Disease

**DOI:** 10.3389/fimmu.2014.00036

**Published:** 2014-02-05

**Authors:** Anca Dorhoi, Marco Iannaccone, Jeroen Maertzdorf, Geraldine Nouailles, January Weiner, Stefan H. E. Kaufmann

**Affiliations:** ^1^Department of Immunology, Max Planck Institute for Infection Biology, Berlin, Germany

**Keywords:** tuberculosis, biomarker, inflammation, microRNA, interferon, neutrophil, chemokine

## Abstract

Tuberculosis (TB) is a major health issue globally. Although typically the disease can be cured by chemotherapy in all age groups, and prevented in part in newborn by vaccination, general consensus exists that development of novel intervention measures requires better understanding of disease mechanisms. Human TB is characterized by polarity between host resistance as seen in 2 billion individuals with latent TB infection and susceptibility occurring in 9 million individuals who develop active TB disease every year. Experimental animal models often do not reflect this polarity adequately, calling for a reverse translational approach. Gene expression profiling has allowed identification of biomarkers that discriminate between latent infection and active disease. Functional analysis of most relevant markers in experimental animal models can help to better understand mechanisms driving disease progression. We have embarked on in-depth characterization of candidate markers of pathology and protection hereby harnessing mouse mutants with defined gene deficiencies. Analysis of mutants deficient in miR-223 expression and CXCL5 production allowed elucidation of relevant pathogenic mechanisms. Intriguingly, these deficiencies were linked to aberrant neutrophil activities. Our findings point to a detrimental potential of neutrophils in TB. Reciprocally, measures that control neutrophils should be leveraged for amelioration of TB in adjunct to chemotherapy.

## Introduction

Until today, segregation between basic and applied research has not been fully overcome in medical science, including immunology. Principally, basic research is conceived as hypothesis-driven, which benefits from choice of the most suitable experimental approach. Successful studies end with an outlook on medical application. In rare instances when this outlook indeed materializes, the whole endeavor is considered a perfect example of translational medicine. In contrast, applied clinical research is hampered by various layers of complexity, including heterogeneity of human populations and limitations in experimental approaches.

**Figure 1:**
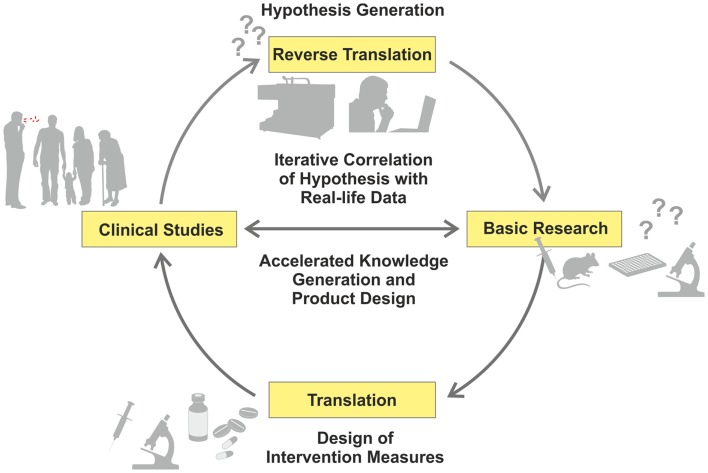
**Iterative cycle between translation and reverse translation in TB research and development**. Canonical science frequently follows translational pathways from basic research to clinical studies (pro for basic research as starting point: hypothesis-driven proof of principle; con: dissociation of hypothesis from real-life). In this way, proof of principle derived from basic research can be validated under real-life conditions. Increasingly, reverse translation of clinical observations into hypothesis generation is pursued (pro for clinical studies as starting point: reality-driven hypothesis generation; con: generation of questions without direct answers). Clinical studies and trials generate “real-life” data, which are subsequently tested and verified by basic research. This allows for validation of reality-driven questions raised in clinical studies. An iterative correlation of hypothesis-driven and data-driven research in the clinical context, with the help of computational analyses, will accelerate both generation of knowledge and design of novel intervention measures.

Recent advances in “omics” (i.e., high-throughput, HT, approaches, such as genomics or transcriptomics) have allowed a reciprocal strategy related to data-driven and hypothesis-generating approaches, which have been recognized as valuable complements to the hypothesis-driven path (1). Comparative studies of patients and healthy controls using different omics readouts allow a deeper understanding of mechanisms underlying disease progression, and identification of thus far unknown or insufficiently understood biological functions (2, 3). Hence, new research questions can be formulated, based on findings in the clinical context, which can subsequently be dissected in appropriate experimental models (Figure 1) – in short, from the bed to the bench (4).

Immunology of tuberculosis (TB) is a case in point: mechanisms underlying pathology and protection in TB are highly intertwined and quantitative rather than qualitative differences tip the balance toward disease progression (5). We have embarked on analyzing biomarkers that distinguish TB patients from healthy individuals and identified markers, which have fueled our interest to better understand their biological functions in TB.

## The Issue: Tuberculosis

With a morbidity of 8.6 million cases and a mortality of 1.3 million deaths annually, TB remains a major health issue, surpassed only by human immunodeficiency virus/acquired immune deficiency syndrome (HIV/AIDS) (6, 7). In fact, the two diseases have joined forces to create a perfect storm: TB is the major cause of death of HIV-infected individuals and HIV/AIDS the driving force for TB reemergence (6). Infection with *Mtb* transforms into active disease in only 5–10% resulting in 2 billion individuals with latent TB infection (LTBI), who remain healthy. Immunity is orchestrated by T lymphocytes that activate anti-mycobacterial activities in infected macrophages (5, 8). This is a local event focused on granulomatous lesions. *Mtb* is efficiently contained in solid granulomas where it adopts a dormant stage characterized by low replication rate and minimal metabolic activity (9, 10).

Globally, the ca. 2 billion healthy individuals with LTBI maintain this stage by means of an active immune response (5, 8). Yet, once it deviates, active disease develops. This is preceded by increasing necrosis of the granuloma followed by liquefaction. In the caseous granuloma, *Mtb* is resuscitated and resumes high replicative and metabolic activity (9, 10). Often in TB patients, granulomas of different maturation stages coexist (11). Whilst in some granulomas the immune response is still capable of controlling *Mtb*, in others, it has already failed and *Mtb* has mastered the battle field (9, 10). Divergent immune activities in different lesions create major obstacles in the analyses of relevant immune responses in the periphery: it is likely that the circulating leukocytes reflect an averaged dominant stage of immune defense, but not unique activities operative in different lesions of varying immune status.

In infants, serious forms of TB can be prevented by vaccination. The vaccine, bacille Calmette–Guérin (BCG), therefore is still part of vaccination programs in countries where TB is endemic (12). Today, BCG is the most widely used vaccine with at least 4 billion total, and ca. 100 million annual, administrations. Yet, an efficacious vaccine against pulmonary TB in all age groups, which is not only the most prevalent form, but also the major source of transmission, is not available. Currently, novel vaccine candidates are being developed of which more than a dozen are undergoing clinical evaluation (13).

Tuberculosis can be cured by chemotherapy. However, successful treatment is long-lasting, requiring three to four drugs given over a period of at least 6 months. Not the least due to the complex and long-lasting treatment regimen, resistant TB is on the rise (14–16). Fifty million individuals are infected with multidrug-resistant (MDR)-*Mtb* strains that cannot be treated adequately with first-line drugs. Therefore, for half a million new MDR-TB cases annually, second-line drugs requiring longer and more complex treatment schedules with higher side effects must be employed. The ca. 50,000 individuals registered in 92 countries, who have developed extensively drug-resistant (XDR)-TB, are even worse off since this form of TB is almost untreatable (6). In the meantime, totally drug-resistant (TDR)-TB has been notified in countries in Asia, Europe and Africa. No drugs are available to treat this form of TB. As a corollary, cost for TB control is increasing. It has been estimated that the European Union invests more than half a billion € for TB control and that the real cost including loss of human capital is in the order of 6 billion € (17). Globally, somewhere between 20 and 200 billion US$ are lost due to TB, resulting in 0.5% loss of gross national income.

The epidemiologic facts of 1.3 million deaths and 8.6 million new cases of TB annually, may be viewed as perplexing in the face of 2 billion healthy individuals with LTBI. This conundrum immediately raises the question: what is the difference between individuals who successfully control one of the most devastating pathogens, whilst others succumb? This enigma is difficult to study in experimental animal models, which, aside from non-human primates, do not reflect the dissociation between LTBI and active TB disease adequately. At the same time, modern HT gene expression profiling of peripheral blood leukocytes could allow a gateway toward the elucidation of gene products involved in resistance underlying LTBI and pathology present in active TB. We launched a critical analysis of biomarkers that distinguish patients with active TB from healthy individuals with LTBI and selected markers of interest for in-depth analyses in experimental mouse models.

## Biosignatures, Biomarkers and Reverse Translation

The past decade has witnessed increasing interest in biomarker research in the area of TB. The potential of these biomarkers ranges from differential diagnosis to predictive response to therapy and risk to disease progression (2, 3). Biomarkers will also play a crucial role in future vaccine and drug trial design (18–20). On top of this, omics marker research can provide a wealth of information, which can be further exploited to decipher underlying resistance and disease mechanisms.

Principally, biomarker research is set in a clinical context and seeks to answer questions such as: How to diagnose or predict disease? How to monitor treatment outcome? How to predict vaccine efficacy and safety? Thus, large-scale HT platforms have been launched with the goal of screening thousands of genes or hundreds of metabolites [for a review see Ref. (2)]. From these studies the view emerges that quantitative biosignatures, rather than individual, “on-off” biomarkers are informative predictors of disease progression, treatment outcome or vaccine efficacy/safety. Basically, mere presence or absence of a single gene in a biosignature is insufficient to achieve error rates below 10%. Rather, relative expression levels of a set of genes or abundances of a set of metabolites form a more informative multidimensional biosignature. Metaphorically, instead of a single decision maker (i.e., a single biomarker), we view a biosignature as a house of representatives that agree on a mechanism, state of disease, or outcome of an intervention by casting a majority vote.

The primary goal of attempts to define biosignatures remains in the clinical arena. Yet, added to this, the functional interpretation of biosignatures allows precious insights into the pathophysiology of TB, as well as the generation of specific hypotheses to be tested in an experimental setting (3, 21). Vice versa, identifying relevant biological processes can clearly drive the search for useful biomarkers (22). Biomarkers should thus not be viewed as a computational black box that exists outside of biology; rather, they can both drive, and profit from, specific hypothesis-driven research.

Whole genome expression profiling by microarray analysis of peripheral blood cells, which has been applied most widely (23–30), provides a large body of data tempting researchers to formulate novel hypotheses. Prominent in peripheral blood, active TB disease is reflected by an increased activity in interferon (IFN) signaling, mainly in circulating neutrophils (23). In a similar fashion, most studies on blood cells have identified up-regulated signaling through Fc gamma receptors and elevated activity of the complement system [Ref. (23–25, 29, 31), for a review see Ref. (2)]. Finally, several publications describe activation of the Janus kinase-signal transducer and activator of transcription (JAK-STAT) pathway (23–25), and abnormal functionality of regulatory suppressors of cytokine signaling [SOCS3, Ref. (27)] in TB. Such findings point to dysregulation in several key biological pathways, marking chronic immune activation and immune pathology of TB disease. The biomarker studies performed over the past years have revealed several TB-related signatures as described herein. The current challenge is to identify markers that are specific and unique for TB (for a short listing of markers in TB and other pulmonary diseases; see Table 1). For example, the first “TB-specific” signature identified in a comparison with other infectious diseases (23), turned out shortly afterward to be also present in sarcoidosis (26, 32), which has high clinical similarity to TB. Elevated IFN-signaling, Fc gamma receptor signaling and complement activation are also shared with other respiratory diseases (26, 32, 33). A recent study (34) identified blood transcriptome signatures that were identified in both HIV^−^ and HIV^+^ donors. Such findings are encouraging that future TB-specific signatures could also be applied in HIV-endemic countries.

**Table 1:** **Genes identified as differentially expressed in TB compared to healthy controls and reported as a part of a biosignature**.

Interferon signaling	Pattern recognition receptor and inflammation	Neutrophil response	Adaptive immunity	Chemokines and receptors	Complement system	Fc receptors	Other
IFIT2; 3 (s)	TLR5 (s^a^; l)	MPO (p)	BATF2 (s)	CXCR3 (s)	C1QA	CD64 (s; p; l)	RAC1 (s)
IFI44L (s)	CD32 (s)	CTSG (s^a^)	CD4	CXCR4 (s)	C1QB (s^a^)	CD32 (s)	SEC14 (s)
GBP1; 2; 5; 6 (s)	IRAK1; 3; 4 (s)	LTF (s; p)	CD40 (s)	CXCR5 (s)	C2		KLF2 (s^a^)
OAS1 (s^a^)	ETS2 (s)	BPI (s^a^)	IGHM (s)	CXCL9 (s)	SERPING1 (s)		HIF1A (s)
SOCS1 (s)	NAMPT (s; p)	DEFA4 (p)	IGHD (s)	CXCL10 (s)			HLTF (s)
SOCS5 (s)	CD163 (s; l)	NCF1 (s)	IGJ	CXCL14 (s^a^)			PSMA 1–7 (s)
	TGFB1 (s)	LCN2		CCL23 (s)			UCN2 (s)
	TRAF5	MMP9 (s; l; p)					SMARCD3 (s)
		MMP8 (p)					FOXB1
							FOXC2
							TIMP (s)
							RAB13 (s^a^; p)
							RAB33 (s)
							CASP8 (s)

*Annotations in brackets denote whether a gene has also been reported as differentially expressed in another disease condition by Maertzdorf et al. (26) or Bloom et al. (32); s, sarcoidosis; p, pneumonia; l, lung cancer*.

*^a^Denotes genes that, while identified as differentially expressed in sarcoidosis, differ significantly in their expression from TB*.

Although signatures, as described above, are related to elevated anti-microbial activities of the immune response, they can also have detrimental effects. One informative example is the preponderant role of IFNs in the inflammation sustained during TB disease. While the role of type II IFN (IFN II or IFN-γ) in protection against TB is well established (35, 36), the role of type I IFN (IFN I) is less clear. Rather IFN I responses have been generally associated with anti-viral defense (37). Yet, both IFN I and IFN II signaling pathways are markedly up-regulated in TB. This led several groups to embark on analysis of IFN I in experimental TB and investigate interference between IFN I and II in mycobacterial infections.

## IFN I Cross-Regulates Cytokine Networks in TB

Non-redundant functions have been attributed to IFN I and II during pulmonary TB in TB-resistant mice (38). Both cytokines contribute to adequate differentiation, survival and/or recruitment of myeloid cells to the lungs early during infection. Anti-inflammatory roles affecting leukocyte recruitment were uncovered in animals with intact IFN I signaling. These observations, however, contrast data obtained with animals receiving IFN I inducers (39), which develop exacerbated lung inflammation. Others have proposed that similarly to IFN II, IFN I limits availability of IL-1, a key pro-inflammatory cytokine (40, 41). The anti-inflammatory roles of IFN I in murine TB, as described for TB-resistant mice, are partially in line with results recorded for acute bacterial infections (42). Yet, these effects are opposed to those reported for chronic insults and infections (43, 44), which are characterized by IFN I-triggered inflammation. These apparently contradictory findings suggest biphasic and perhaps context-specific activity of IFN I. In support of this notion, investigations focusing on lymphocytic choriomeningitis virus (LCMV) infection describe a paradoxical detrimental role of IFN I during the chronic phase of this viral disease (45, 46).

In addition, cross-regulation between IFN I and II was revealed during mycobacterial infection, thereby explaining, to some extent, divergent observations. Via IL-10, IFN I regulates expression of the receptor for IFN II and subsequently cellular responsiveness to protective IFN-γ (47). On the other hand, absence of the IFN I-inducible ubiquitin-like intracellular protein ISG15 limits release of IFN II (48). It appears that both positive and negative regulation loops between IFN I and II exist and additional regulatory check points will likely be identified in the future. Although considerable progress has been achieved to streamline the biomarker value of IFN I, there is still need to decipher in greater detail local and perhaps systemic effects of IFN I signaling in TB.

## miR-223 Fine-Tunes Inflammation in TB

In the beginning, gene expression profiling mainly targeted protein-coding genes. More recently, the potential value of microRNAs (miRs), as TB biomarkers, has gained increasing interest (49) (Table 2). miRs have a profound impact on the biological activity of proteins by regulating messenger RNA (mRNA) stability and translation (50). Parallel analysis of miRs and mRNAs revealed significant correlations between expression of protein-coding and regulatory small miRs, suggesting functional relevance in TB (26). Particular miRs have been tentatively identified as potential biomarkers based on their differential expression levels (51–55). Moreover, by means of HT analyses of TB mRNAs and miRs, we identified clusters of correlated miRs and mRNAs, which were differentially expressed between TB and controls (2, 26) and enriched for immune-related functions. Their direct biological function in TB, however, remains largely elusive. Whereas miRs are generally considered to fine-tune mRNA expression rather than performing unique functions, we recently demonstrated a unique biological role of one such miR in inflammatory processes in TB (56).

**Table 2:** **Candidate microRNA biomarkers for active TB**.

Sample type	microRNA	Reference
Monocytes	hsa-mir-582-5p	(57)
CD4^+^ T cells	has-miR-21, has-miR-26a, has-miR-29a, and miR-142-3p	(52)
Serum	hsa-miR-361-5p, hsa-miR-889, and miR-576-3p	(53)
PBMCs	hsa-miR-146a and has-miR-424	(58)
Sputum	hsa-mir-3179, has-miR-147, and hsa-miR-19b-2-5p	(55)
Peripheral whole blood	hsa-miR-144	(26)
PBMCs	hsa-miR-155 and hsa-miR-155-3p	(59)
PBMCs	hsa-miR-144, hsa-miR-365, hsa-miR-424, and hsa-miR-451	(54)
Serum	hsa-miR-29a	(51)

In our studies, we focused our attention on miR-223, which had been identified in one study as one of the most up-regulated miRs in peripheral blood of patients with active TB compared to individuals with LTBI (54). MiR-223 expression is induced during granulopoiesis (60), controlled by different myeloid transcription factors (61, 62), and reaches its highest level in mature neutrophils. Additionally, miR-223 modulates cell activation by targeting NLR family pyrin (NLRP) containing domain 3 (NLRP3) inflammasome and I-kappa-B-kinase (IKK) alpha (IKK-α) (63, 64). To address the biological role of miR-223 during TB, we employed the aerosol *Mtb* infection model of miR-223 mutant mice (60). MiR-223-deficient mice were highly susceptible to pulmonary TB. Lethality was preceded by profound alteration of the lung structure, high bacterial burden, and exacerbated inflammation, which was mostly due to uncontrolled neutrophil migration to the site of infection. Lung gene expression profiling highlighted genes involved in neutrophil recruitment and the immune response as potential targets for miR-223.

Corroboration of gene expression profiles with predicted targets of miR-223, combined with molecular investigations, allowed us to identify chemokine (C-X-C motif) ligand 2 (CXCL2), chemokine (C-C motif) ligand 3 (CCL3), and interleukin 6 (IL-6) as novel targets of miR-223. These proteins were abundant during *Mtb* infection and directed neutrophil activation, and/or recruitment. Indeed, CXCL2 and CCL3 were first identified as chemotactic molecules for neutrophils produced by activated macrophages while IL-6 regulates granulopoiesis (65–67). Thus, in our model of experimental TB, the absence of miR-223 was responsible for uncontrolled expression of chemotactic mediators, CXCL2 and CCL3, and heightened neutrophil availability due to impaired granulopoiesis as a consequence of uncontrolled IL-6 expression. These conditions, concurrent with more abundant chemokine (C-X-C motif) receptor 2 (CXCR2) expression (60), were responsible for impaired neutrophil migration to the lung during TB and consequently tissue destruction.

These results add further knowledge to the role of miRs during TB and in particular suggest that miR-223 controls TB susceptibility by limiting neutrophil recruitment through regulation of pro-inflammatory chemokines.

## Chemokine-Dependent Neutrophil Influx Modulates TB Pathology and Susceptibility

Our biomarker analysis focusing on miR-223 in experimental TB of mice as well as reports by others reveal a profound correlation between disease susceptibility, TB pathology, and magnitude of the neutrophil responses. DBA/2, CBA/J, and C3HeB/FeJ, all *Mtb*-susceptible mouse strains, suffer from increased pulmonary neutrophil influx (68–70). Treatment of these animals with anti-inflammatory agents alone or combined with anti-mycobacterial chemotherapy limits progression of active TB (70, 71), suggesting that targeting neutrophilic inflammation is a valid option for cure of TB. Likewise, susceptible gene-deletion mutant strains, such as *Card9*^−/−^ and *miR-223*^−/−^ mice can be rescued by antibody-mediated neutrophil depletion (56, 72). In patients with pulmonary TB, neutrophils are abundant in BAL fluid and sputum and show higher bacterial burden than macrophages (73). The potential beneficial role of neutrophils in TB through killing the pathogen remains a matter of debate. Reports on a role of neutrophils in human TB range from assigning them solely phagocytic capacities to *Mtb*-killing capacities (74). In the zebrafish model, neutrophils kill *M. marinum* by engulfing-infected macrophages (75). In mice, immune-advantageous functions of neutrophils in TB have been linked to dendritic cell migration and T cell priming (76, 77).

Neutrophils can rapidly enter sites of inflammation under the direction of humoral factors including chemokines, notably of the ELR^+^ CXC family, which bind to CXCR1 and CXCR2. ELR^−^ CXC chemokines, for example, CXCL13, are primarily chemoattractants for lymphocytes and bind to CXCR3, CXCR4, CXCR5, and CXCR7. Further subfamilies, include the CC chemokine subfamily, which comprises various members and bind to CC receptors; their target cells include most types of leukocytes. The third and fourth chemokine subgroups contain few members: the (X)C chemokine family consists of XCL1 and XCL2, and both primarily target T cells expressing the receptor XCR1. The CX_3_C chemokine subfamily comprises to date only one known member, CX3CL1, which serves in its soluble form as chemoattractant for T cells and monocytes and in its cell-bound form as adhesion molecule [reviewed in Ref. (78, 79)].

To better understand how excessive neutrophil influx participated in increased TB susceptibility, we embarked on studying the role of CXCR2/CXCL5 in neutrophil control. Different chemokine/chemokine receptor pairs and inflammatory mediators are described to be involved in neutrophil influx into the lung (80). However, we and others had noted that the neutrophil-chemotactic receptor, CXCR2 and its ligand, CXCL5 [or LPS-induced CXC chemokine (LIX)] are strongly up-regulated early following *Mtb* infection (69, 78, 81). Other ligands of CXCR2 comprise ELR^+^ chemokines, such as CXCL1 [or keratinocyte-derived chemokine (KC)], CXCL2 [or macrophage inflammatory protein 2 (MIP-2)], and CXCL15 (or lungkine).

The potential of chemokines and their receptors in shaping immune responses against *Mtb* have thus far focused on lymphocyte recruitment and granuloma organization (78). The influence of the chemokine system on neutrophil responses and inflammation in TB is poorly characterized. *CXCR2*^−/−^ mice show normal pulmonary neutrophil influx following intratracheal infection with an atypical *Mycobacterium, M. avium*. After intraperitoneal challenge with *M. avium*, however, neutrophil recruitment was impaired in *CXCR2*^−/−^ mice albeit without affecting bacterial burden (82). We found that following aerosol *Mtb* infection, neutrophil influx into alveolar spaces depended on CXCR2 as well as CXCL5. Moreover, absence of either CXCR2 or CXCL5 rendered mice more tolerant to high-dose *Mtb* infection (83).

Since multiple chemokines can bind CXCR2, they have generally been regarded as redundant. However, kinetic analyses indicate their temporal regulation (78) and cell type-specific expression of different chemokines points toward their spatial regulation. In TB, lung epithelial cells served as the pulmonary source of CXCL5 (83). Neutrophil-attracting chemokines including CXCL2 and CXCL1 are abundantly secreted by macrophages and neutrophils. CXCL1, but not CXCL2, is also secreted by pneumocytes. CXCL15 is solely secreted by bronchial epithelial cells (80, 84). CXCL5 is produced by platelets and various tissue-resident cells, such as alveolar epithelial cells (85, 86), cardiac myocytes (87), enterocytes (88), and aortic endothelial cells (89).

Taking the pathologic potential of dysregulated neutrophil responses into account, it appears essential that the host employs mechanisms to tightly regulate their recruitment into sensitive tissues. We propose that one mechanism is the temporal and spatial regulation of neutrophil-attracting chemokines. Thus, rather than being redundant, we suggest that neutrophil-attracting chemokines targeting the same receptor express unique, often additive effects.

## Concluding Remarks

Tuberculosis biomarker research continues to generate signatures with clinical applicability and additionally furnishes novel hypotheses related to disease pathophysiology. We followed several candidate pathways and molecules that emerged from transcriptomics studies, including miR-223. Murine experiments provided deeper insights into disease processes influenced by this molecule. Neutrophils represented key effector cells of pathogenesis. In a complementary approach, we investigated the pathways that control neutrophil recruitment to the lung in progressive TB and distinguished a hitherto unappreciated relevance of a unique chemokine, namely CXCL5.

These examples illustrate that reverse translation is a valid approach and perhaps most importantly that iterative cross-examining of basic research findings and patient “omics” data, allows novel insights into TB pathogenesis. We envisage that additional aspects of disease pathophysiology will be uncovered by integrating information from multiple patient-driven HT studies, such as metabolomics, proteomics, lipidomics along with transcriptomics, and deep-sequencing. In a further step, host information could be complemented by pathogen screens. This strategy will facilitate insights into host–pathogen interplay and allow prediction of interaction algorithms to be experimentally validated. As TB vaccine research and development suffer from lack of rigorous correlates of protection, we propose that reverse translation can significantly contribute to better understanding of basic mechanisms underlying pathophysiology. Such an approach will not only form the basis for identification of biosignatures that predict risk of disease but also predict vaccine efficacy. In a similar vein, biosignatures derived from TB vaccine trials can provide novel insights into vaccine-induced protective mechanisms. The feasibility of such an approach has proven successful in trials with licensed vaccines (18). Reciprocally, experimental TB research can facilitate rational design of novel intervention measures.

## Author Contributions

All have contributed to this work (It is not original work.): Anca Dorhoi, Marco Iannaccone, Jeroen Maertzdorf, Geraldine Nouailles, January Weiner 3rd, Stefan H. E. Kaufmann. Drafting the work or revising it critically for important intellectual content: Anca Dorhoi, Marco Iannaccone, Jeroen Maertzdorf, Geraldine Nouailles, January Weiner 3rd, Stefan H. E. Kaufmann. Final approval of the version to be published: Anca Dorhoi, Marco Iannaccone, Jeroen Maertzdorf, Geraldine Nouailles, January Weiner 3rd, Stefan H. E. Kaufmann. Agreement to be accountable for all aspects of the work: Anca Dorhoi, Marco Iannaccone, Jeroen Maertzdorf, Geraldine Nouailles, January Weiner 3rd, Stefan H. E. Kaufmann.

## Conflict of Interest Statement

Stefan H. E. Kaufmann is coinventor of VPM1002. The other co-authors declare that the research was conducted in the absence of any commercial or financial relationships that could be construed as a potential conflict of interest.
